# The Auditory Pathway in Congenitally Cytomegalovirus-Infected Human Fetuses

**DOI:** 10.3390/ijms25052636

**Published:** 2024-02-24

**Authors:** Liliana Gabrielli, Maria Paola Bonasoni, Giulia Piccirilli, Evangelia Petrisli, Simona Venturoli, Alessia Cantiani, Matteo Pavoni, Concetta Marsico, Maria Grazia Capretti, Giuliana Simonazzi, Tiziana Lazzarotto

**Affiliations:** 1 Microbiology Unit, IRCCS Azienda Ospedaliero-Universitaria di Bologna, 40138 Bologna, Italy; liliana.gabrielli@aosp.bo.it (L.G.); evangelia.petrisli@aosp.bo.it (E.P.); simona.venturoli@aosp.bo.it (S.V.); tiziana.lazzarotto@unibo.it (T.L.); 2 Pathology Unit, Azienda USL-IRCCS di Reggio Emilia, 42123 Reggio Emilia, Italy; mariapaola.bonasoni@ausl.re.it; 3 Section of Microbiology, Department of Medical and Surgical Sciences, University of Bologna, 40138 Bologna, Italy; alessia.cantiani@studio.unibo.it (A.C.); matteo.pavoni2@unibo.it (M.P.); 4 Neonatal Intensive Care Unit, IRCCS AziendaOspedaliero-Universitaria di Bologna, 40138 Bologna, Italy; conce.marsico@gmail.com (C.M.); mariagrazia.capretti@aosp.bo.it (M.G.C.); 5 Obstetric Unit, IRCCS Azienda Ospedaliero-Universitaria di Bologna, 40138 Bologna, Italy; giuliana.simonazzi@unibo.it; 6 Section of Obstetrics, Department of Medical and Surgical Sciences, University of Bologna, 40138 Bologna, Italy

**Keywords:** cytomegalovirus, congenital infection, hearing loss, temporal lobe, auditory cortex, inner ear, microglia

## Abstract

Congenital cytomegalovirus (CMV) infection is the main cause of non-hereditary sensorineural hearing loss (SNHL). In order to shed light on SNHL pathophysiology, we examined the auditory pathway in CMV-infected fetuses; the temporal lobe, in particular the auditory cortex, and the inner ear. We investigated both inner ears and temporal lobes of 20 human CMV-infected fetuses at 21 weeks of gestation. As a negative group, five fetuses from spontaneous miscarriages without CMV infection were studied. Inner ears and temporal lobes were histologically examined, immunohistochemistry for CMV and CMV-PCR were performed. On the auditory cortex, we evaluated the local microglial reaction to the infection. CMV-positive cells were found in 14/20 brains and the damage was classified as severe, moderate, or mild, according to histological features. Fetuses with severe brain damage had a statistically higher temporal lobe viral load and a higher number of activated microglial cells in the auditory cortex compared to fetuses with mild brain damage (*p*: 0.01; *p*: 0.01). In the inner ears, the marginal cells of the stria vascularis were the most CMV positive. In our study, CMV affected the auditory pathway, suggesting a tropism for this route. In addition, in the auditory cortex, microglial activation may favor further tissue damage contributing to hearing loss.

## 1. Introduction

Congenital cytomegalovirus (CMV) infection is the leading non-genetic cause of sensorineural hearing loss (SNHL) and neurodevelopmental delay in children [1,2,3,4]. It accounts for 8–21% of all congenital SNHL at birth, reaching 25% by the age of 4 years due to late-onset hearing loss [5,6,7,8,9].

A particular feature is fluctuating hearing loss that is not explained by concurrent middle ear infections occurring at only a few unilateral or bilateral frequencies [10]. Further deterioration or progression in hearing loss can be observed in about half of the children with SNHL at birth, both in children with symptomatic or asymptomatic infection, although symptomatic children have a greater degree of severity and earlier progression of hearing loss [10]. Without neonatal routine screening programs, asymptomatic newborns are not diagnosed with congenital CMV infection; however, 10–15% of them develop long-term sequelae, especially isolated SNHL [11]. For many children, without a proper diagnosis at birth, their hearing impairments will never be related to congenital CMV due to the subtlety of clinical presentation and the difficulties in providing a late diagnosis (CMV detection in Guthrie card) [10].

CMV neurotropism is well known and ultrasound (US) and MRI are the gold standard imaging tools in the prenatal setting to detect congenital infection. Fetal cerebral US findings, with variable incidence and severity, include microcephaly, ventriculomegaly, parenchymal calcifications, typical calcifications in the lenticulostriate vessels in the thalami, hyperechoic periventricular halo, and subependymal cysts. Especially in the second trimester, progressive ventricular enlargement (>20 mm) associated with periventricular hyperechogenicity should warrant further investigation. Abnormal gyration, such as lissencephaly and polymicrogyria, may be recognized in severe cases as an altered thickness of the cortex. However, MRI is more sensitive in detecting the more subtle migration anomalies, particularly located in the temporal lobes, such as heterotopias and polymicrogyria. The latter is characterized by excessive cortical infoldings for gestational age, and it is a consequence of injuries occurring between the 18th and the 24th week of gestation. White matter T2 hyperintensity, though highly subjective, has been reported in temporal lobes, also in association with a globally reduced volume [9,12].

Histological studies, carried out in congenitally infected fetuses and children, have shed light on the pathophysiology of CMV infection [13,14,15,16,17,18,19]. CMV-positive cells might be found in any cerebral region (leptomeninges, cortex, white matter, germinal matrix, or grey matter) and any kind of cells might be infected (neurons, neuroblasts, glia, endothelial, ependymal, and meningeal). Especially in fetuses with severe cerebral injuries [14,16], cortical damage included laminar necrosis, in which the third layer was wiped out and replaced by macrophages, and early polymicrogyria, with abnormal infoldings of the developing sulci. In the white matter, periventricular leukomalacia, capillary proliferation with plump endothelium, glial karyorrhexis, macrophage infiltration, microglial nodules with activated T-lymphocytes, and ferruginated neurons (i.e., deposition of iron and calcium in neuronal cell body, axons, and dendrites) were the most common neuropathological features. Fetal cerebral damage is thus a combination of multiple injuries. Remarkably, CMV has been found to have a preferential tropism for neural stem/progenitor cells and neuronal committed cells, leading to impaired migration resulting in lissencephaly and/or polymicrogyria [16].

The anatomical counterpart of clinical SNHL in congenital CMV infection has also been documented in different studies [15,17,20].

The inner ear contains the vestibular apparatus, which is the organ of balance, and the cochlea, the organ of hearing. The auditory pathway starts in the cochlea, then the cochlear nerve (VIII nerve) transmits information to the cochlear nuclei located in the medulla. Eventually, the impulse travels upward through the superior olivary complex in the pons and the inferior colliculus in the midbrain. This structure projects via the medial geniculate nucleus in the thalamus to the auditory cortex in the superior temporal lobe [21,22,23]. This cortical area, located bilaterally at the upper sides of the temporal lobes, processes auditory information taking part in the spectro-temporal analysis of the inputs passed on from the ear, interpreting the time and frequency of the signals [23]. Cerebral lesions due to infections in the auditory cortex are generally uncommon, but damage in this cerebral area can result in different clinical deficits as the left auditory cortex processes temporal data, and the right auditory cortex processes spectral information [24,25].

The aim of our study was to increase knowledge in the pathophysiology of SNHL in congenitally infected fetuses. We examined the terminal parts of the auditory pathway: the inner ear and the temporal lobe, in particular the auditory cortex. In this area, we evaluated the presence of residential rod microglia, as this is known to play a key role in neuroinflammation [26].

## 2. Results

The bilateral auditory pathway, comprising the temporal lobe, including the auditory cortex, and the inner ear, of 19 fetuses at 21–22 weeks of gestational age were histologically and virologically examined. A total of 14 out of 19 fetuses had a congenital CMV infection documented at 21 weeks of gestation by prenatal diagnosis, as the viral load in amniotic fluid was more than 10^5^ copies/mL. All mothers had a primary CMV infection arising before the 12th week of gestation. In total, 5 out of 19 fetuses from CMV seronegative women were also studied as control cases. Three of them were a termination of pregnancy due to fetal cardiac malformation and two were spontaneous miscarriages caused by cervical incompetence.

### 2.1. Temporal Lobe

In all the 14 fetuses with congenital CMV infection, the immunohistochemistry showed CMV-positive cells in both temporal lobes. According to histological features, the cerebral damage was classified as severe in three, moderate in seven, and mild in four (Table 1).

In this cerebral area, CMV-PCR showed a viral load range of 158–395 copies/5 ng in fetuses with severe brain damage, 80–238 copies/5 ng in moderate cases, and 14–33 copies/5 ng in mild cases. Fetuses with severe and moderate brain damage had a statistically significant higher viral load compared to fetuses with mild (*p*: 0.01; *p*: 0.002), respectively. No statistically significant difference was observed between fetuses with severe and moderate brain damage (*p*: 0.09). No CMV-positive cells and CMV-DNA were observed in the five control fetuses.

**Figure 1:**
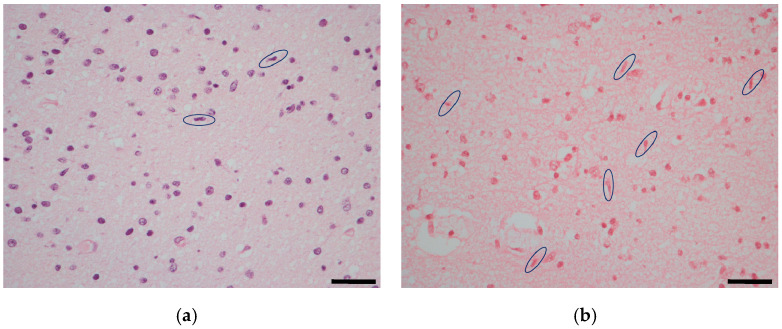
Microglial rod-shaped cells (circled) in the auditory cortex: a control case (**a**) compared to a CMV congenitally infected fetus with moderate brain damage (**b**) hematoxylin and eosin staining. Magnification: 40 HPF, scale bar 50 µm.

In order to evaluate the local microglial reaction to CMV infection, the number of rod-shaped microglial cells was counted in the auditory cortex. The range was 90–208 cells in fetuses with severe brain damage, 44–190 cells in moderate cases, and 33–35 cells in mild cases, respectively. In the control group, the number of cells was within the range of 10–30 (Figure 1). Fetuses with severe and moderate brain damage had a statistically significant higher number of microglial cells compared to fetuses with mild brain damage (*p*: 0.01; *p*: 0.01), respectively. No statistically significant difference was observed between fetuses with severe and moderate brain damage (*p*: 0.3),or between fetuses with mild brain damage and the control group (*p*: 0.06).

### 2.2. Inner Ear

CMV-positive cells were found in 9 out of 14 inner ears, in particular in all 3 fetuses with severe brain damage and in 6/7 with moderate brain damage (Table 1).

**Figure 2:**
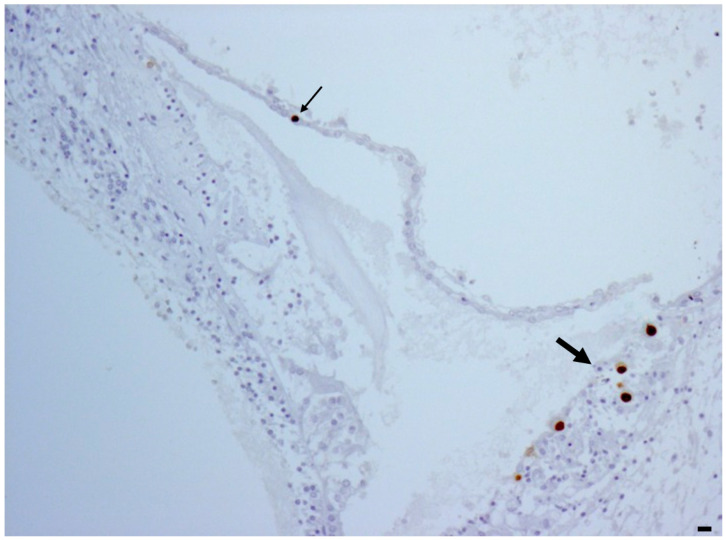
CMV immunohistochemistry showing CMV positivity in the marginal cell layer (big arrow) and in the Reissner’s membrane (small arrow). Magnification: 10 HPF, scale bar 50 µm.

Regarding the cochlea, in all 9 fetuses with a CMV-positive inner ear, CMV-positive cells were observed in the superficial marginal cells of the stria vascularis, the most vascularized area of the cochlea (Figure 2). CMV-positive cells were also found in the Reissner’s membrane in five fetuses and in the sensory cells of the organ of Corti in one fetus.

Regarding inner ear viral load (Table 1), CMV-PCR showed a viral load range of 1000–1675 copies/5 ng in fetuses with severe brain damage, 0–900 copies/5 ng in moderate cases, and negative in mild cases.

Fetuses with severe brain damage had the highest viral load with a statistically significant difference compared to fetuses with moderate brain damage (*p*: 0.002) and mild brain damage (negative PCR) (*p* < 0.001). No statistically significant difference was observed between fetuses with moderate and mild damage (*p*: 0.17).

No CMV-positive cells and CMV-DNA were observed in the five control fetuses.

Detailed data regarding the auditory pathway features in correlation with histological brain damage are shown in Table S1 in Supplementary Materials.

## 3. Discussion

It is well known that CMV-infected fetuses present damage to the neurosensory system including the brain and inner ear [13,14,15,16,20]. To the best of our knowledge, this is the first study in which the terminal parts of the auditory pathway including the temporal lobe, in particular the auditory cortex, and the inner ear were investigated.

Regarding the temporal lobe, we observed that fetuses with severe and moderate brain damage had a statistically significantly higher viral load compared to fetuses with mild brain damage. The finding that the tissue viral load closely correlated with the histological damage has been previously reported considering the whole brain [13,14,16]. This study, evaluating the temporal lobe region only, is in agreement with previous findings, despite the small number of cases.

The auditory cortex is located at the upper side of the temporal lobe. This area is necessary both for the simple detection of sound and for the discrimination of frequency [22]. In this area, we evaluated microglial activated cells presenting a rod-shaped nucleus. Microglial cells are brain-resident myeloid phagocytes and the only innate immune cells in the central nervous system. They react to neuronal insults and pathological cerebral events, producing inflammatory cytokines. Moreover, they are involved in the development of the CNS’s connectivity and synaptic plasticity [27].

Microglia can react to viral pathogen-associated molecular patterns (PAMPs), detecting nucleotides released from damaged neurons, as they secrete more ATP. Microglia is then recruited through the P2Y12 G-protein coupled receptor, which also increases on the cellular surface by two times in the case of viral infection. Interferon (IFN) 1 pathway is also upregulated in activated microglia, especially IFN-stimulated genes (ISGs), contrasting viral replication and favoring viral clearance. On the whole, the proteins produced by ISG expression contrast viruses hampering cellular access, translation, transcription, and particle release. Pattern recognition receptors (PRRs) such as Toll-like receptors (TLRs), cytosolic RNA-sensing RIG-like receptors (RLRs), and DNA identifiers detect viral presence. PRRs stimulates IFN regulatory factor 3 (IRF3) through the TIR domain-containing adaptor, inducing IFN-β (TRIF), mitochondrial antiviral signaling (MAVS), and stimulator of type I IFN genes (STINGs). In activated microglia, viral PAMPs, started by PRRs, produce robust IFN reactions. For example, PRRs, after PAMPs activation, induce IFN-β production, which, in turn, binds to the IFN-α receptor, able to start IRF7 gene transcription. The latter boasts a thorough IFN-1 response and, sustained by an autocrine or paracrine pathway, strongly interferes with viral infection. Microglia bestows STING controlled antiviral function to neurons and induce IFN-1 in astrocytes through TLR3. 

In experimental studies, it was demonstrated that human endothelial cells exhibit a strong IFN-I response against CMV via a cGAS/STING/IRF3 pathway. Therefore, STING is necessary for the first phase of IFN-I production, limiting early CMV replication [28].

Microglia is able to secrete different kind of cytokines. Cellular studies in vitro have found that CMV-infected astrocytes release the monocyte chemoattractant protein 1 (MCP-1), conveying microglia to the infected cells. Then, microglia produces the tumor necrosis factor α (TNFα), interleukin 1β (IL-1β), and interleukin 6 (IL-6), preventing CMV replication in astrocytes [29]. In particular, TNF-α plays a crucial role as a regulator of acute inflammation because it starts the inflammatory cytokine signaling cascades. It is also involved in homeostatic flexibility, maintaining synaptic connection [30]. Another strong pro-inflammatory cytokine is interleukin-1β, which, in a loop of auto-stimulation, monitors neuroinflammation [31].

Chen accurately reviewed the double role of microglia in viral encephalitis, exerting, at the same time, an antiviral and a pro-inflammatory effect [32]. 

Moreover, in CMV-infected microglia, the CXCL10 T lymphocyte chemotactic factor is produced, which enables T-lymphocyte recruitment, that, in turn, secrete IFN-γ, hindering viral replication [33]. Neurotropic viral infection may alter synaptic transmission. However, both microglia and neurons display C3aR, which binds C3 cleavage products. The latter, altogether with C3, induce microglial phagocytosis of the infected neurons, getting rid of the presynaptic ends. This action avoids trans-synaptic viral spreading and blocks altered synaptic communications from infected neurons [34].

As already mentioned, microglia is fundamental in neuroinflammation, a general term that indicates microglia activation, cytokines and chemokines release, chemiotaxis of extracerebral immune cells, and local parenchymal lesions. At the same time, neuroinflammation includes tissue damage, but also tissue repair after the elimination of the noxious agents. At resting, microglia presents a ramified cytoplasmic structure and exert on the brain a homeostatic function, controlling neuronal and synaptic activity. Especially in the developing brain, their phagocytic property plays a key role in synaptic pruning, building specific network connectivity [26].

Rod microglia is a recognized morphology of activation and is observed in neuropathological disorders such as multiple sclerosis and encephalitis. Rod microglia presents an elongated and narrowed soma due to the retraction of planar processes [20]. This type of microglia usually aligns along damaged neurons, their dendrites and axons making a protective barrier in order to preserve uninjured neurons. Rod microglia can act together in long strings, producing a coordinated effect to contrast local insults [27,35].

In our study, the number of rod-shaped microglial cells was counted in the auditory cortex to evaluate their reaction to CMV infection. We observed that fetuses with severe and moderate brain damage had a statistically significantly higher number of activated cells compared to fetuses with mild brain damage. Instead, in fetuses with mild brain damage and controls, no difference was found. This finding may suggest that microglial activation goes along with viral infection. However, we did not observe strings of adjacent microglial cells, but only an increased number of them. The microglial immune reaction within the auditory cortex may be beneficial in terms of viral clearing but, at the same time, their cytokine production and oxidative stress may indirectly damage neurons and synaptic connectivity [26,36]. Pro-inflammatory cytokines, as part of the first antigen non-specific response, can be especially harmful for the cerebral parenchyma [36].

The auditory cortex is the cerebral end of the auditory pathway, which starts in the cochlea, the organ of hearing. In our study, CMV-positive cells were found in all cochleae of fetuses with severe brain damage and in most of fetuses (6/7) with moderate brain damage. The marginal cell layer of the stria vascularis was always CMV-positive. As previously reported, considering that these cells are paramount in maintaining the positivity of endocochlear potential through potassium recycling, CMV infection may alter the ion circulation. This process may damage the ion channels and dissipate the endocochlear potential. Ion channel damage may also be attributed to CMV positivity in the Reissner’s membrane. In only one case, CMV-positivity was observed in the Organ of Corti, likely contributing to the impairment of hair cell functions and the mechano-transduction of sound waves [15,20]. In the cochlea, as observed in the temporal lobe, there was a correlation between CMV load and brain histological damage.

This is the first study in which both terminal parts of the auditory pathway have been investigated in fetuses at the same gestational age. Other studies examined the inner ear and the whole brain in fetuses at different gestational ages without focusing on the temporal lobe only [19,20]. However, in our study, the structures between the inner ear and the auditory cortex were not investigated because their little sizes of fetuses at 21 weeks of gestation did not allow the correct sampling.

In conclusion, the terminal parts of the auditory pathway were affected by CMV infection with a correlation between the tissue viral load and the severity of brain damage. Microglial activation within the auditory cortex may additionally contribute to impairing the auditory pathway, as neuroinflammation, in the aim to stop infection, can favor further tissue damage.

## 4. Materials and Methods

The temporal lobes and inner ears were investigated using: hematoxylin–eosin staining to evaluate the histological damage, immunohistochemistry to identify the presence and distribution of CMV-positive cells, and real-time PCR to detect and quantify the tissue viral load. All the results were the combination of the right and left sides.

After fetal brain removal, petrous bone was identified and accurately dissected from the temporal bone. Then, the inner ears were kept in a weak decalcifier (fixative Gooding Stewart, containing formaldehyde, Bio-Optica, Milan, Italy) for 24–36 h. A tangential section along the insertion of the VIII nerve allowed the correct cochlear paraffin-embedded inclusion.

For immunohistochemistry, an anti-CMV [8B1.2, 1G5.2, 2D4.2] mouse monoclonal primary antibody (product code: 213 M-26; Cell Marque, Rocklin, CA, USA) that reacts with immediate early, delayed early, and late HCMV antigen preparation was used.

The cerebral damage was classified as severe, moderate, or mild, according to histological features as previously described [14,16]. In particular, brain injuries were classified according to a semiquantitative score that evaluated both the extent and severity of damage, as previously applied in other viral encephalitides [37].

The damage was evaluated by integrating the following pathological findings for cortex, white matter, and germinal matrix:

1. Tissue necrosis: 0 = absent; 1 = focal; 2 = diffuse;
2. Microglial Nodules: 0 = absent; 1 = occasional; 2 = scattered; 3 = multiple.

The final score was given, summing each score of the three cerebral areas, from 0 to 15.

Additional inflammatory changes of the cerebral parenchyma were also considered:

1. Microglial activation: 0 = absent; 1 = focal; 2 = diffuse;
2. Astrocytosis:0 = absent; 1 = focal with ramified astrocytes; 2 = diffuse with ramified astrocytes; 3 = 1 or 2 plus astrocyte nuclear clearing—Azheimer’s type II astrocytes—and/or apoptosis;
3. Vascular changes: 0 = no changes; 1 = focal increased number of vessels; 2 = diffuse increased number of vessels, 3 = 1 or 2 plus plump endothelial cells.

The final score was the totality of each individual value (from 0 to 24).

The whole brain damage was then quantified through a scale ranging from 0 to 39. This method was divided into three groups: severe cerebral damage (score 25–39); moderate cerebral damage (score 16–24); and mild cerebral damage (score 0–15).

Microglial-activated cells presenting a rod-shaped nucleus were counted in hematoxylin and eosin staining in 10 fields at 40 HPF. In particular, five fields were from the left side and five were from the right. The final result was the sum of the counted cells. All the histological slides were analyzed by two independent observers. The temporal lobe area was anatomically identified on the unstained slides, dissected from 2 sections (right and left) of 8 microns and placed in a 1.5 mL tube for the deparaffinization procedure, using 160 µL of the deparaffinization solution (Qiagen, Hilden, Germany). For the inner ear, 2 slices of 8 microns (one from the left side and one from the right) were cut from paraffin-embedded blocks. DNA extraction was performed using the QIAsymphony**^®^** SP instrument with the QIAsymphony DSP DNA Mini Kit (Qiagen, Hilden, Germany). Purified DNA was eluted in 50 μL and the contained human DNA (hDNA) was quantified using a real-time PCR assay, Quantifiler**^®^** Human DNA Quantification Kit (Life Technologies, Carlsbad, CA, USA). Five nanograms of hDNA were processed for HCMV-DNA quantification, carried out using a real-time PCR assay, CMV ELITe MGB™ kit (ELITech Group, Turin, Italy). The tissue viral load for the temporal lobe and the inner ear were reported as number of copies/5 ng of hDNA and were the combined result of left and right sides. The lower limit of quantification (LLoQ) was equal to 13 copies/5 ng of hDNA. Positive results below the LLoQ were censored with a value equal to 10 copies/5 ng hDNA [16,38].

### Statistical Analysis

According to data distribution, verified by the Kolmogorov–Smirnov test, the comparison of viral loads and rod-shaped microglial cells between fetuses with different brain damage was performed using Student’s *t* test. Statistical analyses were conducted using GraphPad Prism version 8.0.1 (GraphPad Software, Inc., San Diego, CA, USA) and a *p* value < 0.05 was considered statistically significant.

## Figures and Tables

**Table 1:** The auditory pathway features in correlation with histological brain damage.

		CMV Load (Copies/5 ng DNA)		Rod-Shaped Microglial Cells (n°/10 Fields 40 HPF)
N° cases	Groups	Temporal lobe	Inner ear	Auditory cortex
5	Control	0	0	10–30
4	Mild CD	14–33	0	33–35
7	Moderate CD	80–238	0–900	44–190
3	Severe CD	158–395	1000–1675	90–208

CD = cerebral damage; HPF = high-power field.

## Data Availability

The data presented in this study are available on request from the corresponding author.

## Supplementary Materials

The supporting information can be downloaded at: https://www.mdpi.com/article/10.3390/ijms25052636/s1.

## References

[1] Rivera L.B., Boppana S.B., Fowler K.B., Britt W.J., Stagno S., Pass R.F. (2002). Predictors of hearing loss in children with symptomatic congenital cytomegalovirus infection. Pediatrics.

[2] Kenneson A., Cannon M.J. (2007). Review and meta-analysis of the epidemiology of congenital cytomegalovirus (CMV) infection. Rev. Med. Virol..

[3] Cannon M.J. (2009). Congenital cytomegalovirus (CMV) epidemiology and awareness. J. Clin. Virol. Off. Publ. Pan. Am. Soc. Clin. Virol..

[4] Ross S.A., Kimberlin D. (2021). Clinical outcome and the role of antivirals in congenital cytomegalovirus infection. Antiviral. Res..

[5] Korver A.M.H., de Vries J.J.C., Konings S., de Jong J.W., Dekker F.W., Vossen A.C.T.M., Frijns J.H.M., Oudesluys-Murphy A.M., the DECIBEL Collaborative Study Group (2009). DECIBEL study: Congenital cytomegalovirus infection in young children with permanent bilateral hearing impairment in The Netherlands. J. Clin. Virol..

[6] Goderis J., De Leenheer E., Smets K., Van Hoecke H., Keymeulen A., Dhooge I. (2014). Hearing loss and congenital CMV infection: A systematic review. Pediatrics.

[7] Avettand-Fenoël V., Marlin S., Vauloup-Fellous C., Loundon N., François M., Couloigner V., Rouillon I., Drouin-Garraud V., Laccourreye L., Denoyelle F. (2013). Congenital cytomegalovirus is the second most frequent cause of bilateral hearing loss in young French children. J. Pediatr..

[8] Nance W.E., Lim B.G., Dodson K.M. (2006). Importance of congenital cytomegalovirus infections as a cause for pre-lingual hearing loss. J. Clin. Virol..

[9] Leruez-Ville M., Foulon I., Pass R., Ville Y. (2020). Cytomegalovirus infection during pregnancy: State of the science. Am. J. Obstet. Gynecol..

[10] Fowler K.B., Boppana S.B. (2018). Congenital cytomegalovirus infection. Semin. Perinatol..

[11] Cushing S.L., Purcell P.L., Papaiaonnou V., Neghandi J., Daien M., Blaser S.I., Ertl-Wagner B., Wagner M., Sheng M., James A.L. (2022). Hearing Instability in Children with Congenital Cytomegalovirus: Evidence and Neural Consequences. Laryngoscope.

[12] Diogo M.C., Glatter S., Binder J., Kiss H., Prayer D. (2020). The MRI spectrum of con-genital cyto-megalovirus infection. Prenat. Diagn..

[13] Gabrielli L., Bonasoni M.P., Lazzarotto T., Lega S., Santini D., Foschini M.P., Guerra B., Baccolini F., Piccirilli G., Chiereghin A. (2009). Histological findings in foetuses congenitally infected by cytomegalovirus. J. Clin. Virol..

[14] Gabrielli L., Bonasoni M.P., Santini D., Piccirilli G., Chiereghin A., Petrisli E., Dolcetti R., Guerra B., Piccioli M., Lanari M. (2012). Congenital cytomegalovirus infection: Patterns of fetal brain damage. Clin. Microbiol. Infect..

[15] Gabrielli L., Bonasoni M.P., Santini D., Piccirilli G., Chiereghin A., Guerra B., Landini M.P., Capretti M.G., Lanari M., Lazzarotto T. (2013). Human fetal inner ear involvement in congenital cytomegalovirus infection. Acta Neuropathol. Commun..

[16] Piccirilli G., Gabrielli L., Bonasoni M.P., Chiereghin A., Turello G., Borgatti E.C., Simonazzi G., Felici S., Leone M., Salfi N.C.M. (2023). Fetal Brain Damage in Human Fetuses with Congenital Cytomegalovirus Infection: Histological Features and Viral Tropism. Cell Mol. Neurobiol..

[17] Davis L.E., Johnsson L.G., Kornfeld M. (1981). Cytomegalovirus labyrinthitis in an infant: Morphological, virological, and immunofluorescent studies. J. Neuropathol. Exp. Neurol..

[18] Peña-Alonso R., Navarrete-Navarro S., Ramon-Garcia G., Hernandez-Mote R., Rodriguez-Jurado R. (1996). Cytomegalovirus infection in children: Frequency, anatomopathologic characteristics and underlying risk factors in 1618 autopsies. Arch. Med. Res..

[19] Teissier N., Fallet-Bianco C., Delezoide A.L., Laquerrière A., Marcorelles P., Khung-Savatovsky S., Nardelli J., Cipriani S., Csaba Z., Picone O. (2014). Cytomegalo-virus-induced brain malformations in fetuses. J. Neuropathol. Exp. Neurol..

[20] Teissier N., Delezoide A.L., Mas A.E., Khung-Savatovsky S., Bessières B., Nardelli J., Vauloup-Fellous C., Picone O., Houhou N., Oury J.F. (2011). Inner ear lesions in congenital cytomegalovirus infection of human fetuses. Acta Neuropathol..

[21] Christov F., Nelson E.G., Gluth M.B. (2018). Human Superior Olivary Nucleus Neuron Populations in Subjects With Normal Hearing and Presbycusis. Ann. Otol. Rhinol. Laryngol..

[22] Pickles J.O. (2015). Auditory pathways: Anatomy and physiology. Handb. Clin. Neurol..

[23] Zachlod D., Kedo O., Amunts K. (2022). Anatomy of the temporal lobe: From macro to micro. Handb. Clin. Neurol..

[24] Pillion J.P., Shiffler D.E., Hoon A.H., Lin D.D. (2014). Severe auditory processing disorder secondary to viral meningoencephalitis. Int. J. Audiol..

[25] Kaga K., Kaga M., Tamai F., Shindo M. (2003). Auditory agnosia in children after herpes encephalitis. Acta Otolaryngol..

[26] Woodburn S.C., Bollinger J.L., Wohleb E.S. (2021). The semantics of microglia activation: Neuroinflammation, homeostasis, and stress. J. Neuroinflammation.

[27] Vidal-Itriago A., Radford R.A.W., Aramideh J.A., Maurel C., Scherer N.M., Don E.K., Lee A., Chung R.S., Graeber M.B., Morsch M. (2022). Microglia morphophysiological diversity and its implications for the CNS. Front. Immunol..

[28] Lio C.W., McDonald B., Takahashi M., Dhanwani R., Sharma N., Huang J., Pham E., Benedict C.A., Sharma S. (2016). cGAS-STING Signaling Regulates Initial Innate Control of Cytomegalovirus Infection. J. Virol..

[29] Cheeran M.C., Hu S., Yager S.L., Gekker G., Peterson P.K., Lokensgard J.R. (2001). Cytomegalovirus induces cytokine and chemokine production differentially in microglia and astrocytes: Antiviral implications. J. Neuro-Oncol..

[30] Rauf A., Badoni H., Abu-Izneid T., Olatunde A., Rahman M.M., Painuli S., Semwal P., Wilairatana P., Mubarak M.S. (2022). Neuroinflammatory Markers: Key Indicators in the Pathology of Neurodegenerative Diseases. Molecules.

[31] Swaroop S., Mahadevan A., Shankar S.K., Adlakha Y.K., Basu A. (2018). HSP60 critically regulates endogenous IL-1β production in activated microglia by stimulating NLRP3 inflammasome pathway. J. Neuroinflammation.

[32] Chen Z., Zhong D., Li G. (2019). The role of microglia in viral encephalitis: A review. J. Neuroinflammation.

[33] Cheeran M.C., Hu S., Sheng W.S., Peterson P.K., Lokensgard J.R. (2003). CXCL10 production from cytomegalovirus-stimulated microglia is regulated by both human and viral interleukin-10. J. Virol..

[34] Vasek M.J., Garber C., Dorsey D., Durrant D.M., Bollman B., Soung A., Yu J., Perez-Torres C., Frouin A., Wilton D.K. (2016). A complement-microglial axis drives synapse loss during virus-induced memory impairment. Nature.

[35] Holloway O.G., Canty A.J., King A.E., Ziebell J.M. (2019). Rod microglia and their role in neurological diseases. Semin. Cell. Dev. Biol..

[36] Rocamonde B., Hasan U., Mathieu C., Dutartre H. (2023). Viral-induced neuroinflammation: Different mechanisms converging to similar exacerbated glial responses. Front. Neurosci..

[37] Masliah E., Achim C.L., Ge N., DeTeresa R., Terry R.D., Wiley C.A. (1992). Spectrum of human immunodeficiency virus-associated neocortical damage. Ann. Neurol..

[38] Gabrielli L., Bonasoni M.P., Foschini M.P., Silini E.M., Spinillo A., Revello M.G., Chiereghin A., Piccirilli G., Petrisli E., Turello G. (2019). Histological Analysis of Term Placentas from Hyperimmune Globulin-Treated and Untreated Mothers with Primary Cytomegalovirus Infection. Fetal. Diagn. Ther..

