# Supplementary material for: The Auditory Pathway in Congenitally Cytomegalovirus-Infected Human Fetuses
Source: Int J Mol Sci. 2024 Feb 24;25(5):2636. doi: 10.3390/ijms25052636 (PMC10932391; doi:10.3390/ijms25052636)
Supplement: Supplementary file 1 [file ijms-25-02636-s001.zip › ijms-2821176-supplementary.pdf]

Table S1. The auditory pathway features in correlation with histological brain damage (Full data).

	CMV load (copies/5 ng DNA)		Rod-shaped microglial cells (n°/10 fields 40HPF)
**Groups**	**Temporal lobe**	**Inner ear**	**Auditory cortex**
**Control**	0	0	10
	0	0	27
	0	0	28
	0	0	30
	0	0	29
**Mean (±SD)**	//	//	24.8 (8.3)
**Mild CD**	14	0	33
	33	0	35
	21	0	34
	25	0	35
**Mean (±SD)**	23.2 (7.9)	//	34.3 (1.0)
**Moderate CD**	80	0	44
	147	218	190
	178	900	110
	88	80	98
	238	49	105
	175	230	97
	122	140	82
**Mean (±SD)**	146.8 (55.7)	231 (306.9)	103.7 (44.0)
**Severe CD**	158	1100	90
	203	1000	208
	395	1675	122
**Mean (±SD)**	252 (125.8)	1258.3 (364.2)	140 (61.0)

SD: Standard Deviation
